# Genetic Background Modulates lncRNA-Coordinated Tissue Response to Low Dose Ionizing Radiation

**DOI:** 10.1155/2015/461038

**Published:** 2015-02-23

**Authors:** Jonathan Tang, Yurong Huang, David H. Nguyen, Sylvain V. Costes, Antoine M. Snijders, Jian-Hua Mao

**Affiliations:** Life Sciences Division, Lawrence Berkeley National Laboratory, 1 Cyclotron Road MS977, Berkeley, CA 94720, USA

## Abstract

Long noncoding RNAs (lncRNAs) are emerging as key regulators of diverse cell functions and processes. However, the relevance of lncRNAs in the cell and tissue response to ionizing radiation has not yet been characterized. Here we used microarray profiling to determine lncRNA and mRNA expression in mammary glands of BALB/c and SPRET/EiJ mice after low-dose ionizing radiation (LDIR) exposure. We found that unirradiated mammary tissues of these strains differed significantly in baseline expressions of 290 lncRNAs. LDIR exposure (10 cGy) induced a significant change in the expression of many lncRNAs. The vast majority of lncRNAs identified to be differentially expressed after LDIR in either BALB/c or SPRET/EiJ had a significantly correlated expression pattern with at least one LDIR responsive mRNA. Functional analysis revealed that the response to LDIR in BALB/c mice is highly dynamic with enrichment for genes involved in tissue injury, inflammatory responses, and mammary gland development at 2, 4, and 8 weeks after LDIR, respectively. Our study demonstrates that genetic background strongly influences the expression of lncRNAs and their response to radiation and that lncRNAs may coordinate the tissue response to LDIR exposure via regulation of coding mRNAs.

## 1. Introduction

Ionizing radiation is a well-known carcinogen in humans, and breast is one of the most sensitive organs to radiogenic cancer [1]. The rate of breast cancer in postwar Japan was among the lowest in the world, but breast cancer contributed a disproportionately large fraction of the radiation-related cancer burden in atomic bomb survivors [2, 3]. The data from the Hiroshima and Nagasaki survivors provides strong evidence for increased breast cancer following single acute doses of 20 cGy and linearity with increasing dose [3–6]. Also, an increase in the incidence of breast cancer has been observed in areas affected by the Chernobyl accident, which resulted in radioactive contamination of large areas of Belarus and Ukraine [7]. A twofold increase in risk was observed when comparing the most (>40 mSv cumulative dose) and least contaminated regions. Interestingly, the increase appeared 10 years after exposure and was most prominent in women exposed at younger age.

More than 50,000 women in the United States have been treated with chest radiation (≥20 Gy) for a pediatric or young adult cancer. Children treated from cancer with radiotherapy have a 2.9 relative risk of subsequent malignancy compared to those who were not [8, 9]. A systematic review of 14 studies concluded that risk of breast cancer increased as early as 8 years following chest radiation and did not plateau with increasing length of follow-up [10]. Studies estimating low-dose radiation-induced cancer risk from diagnostic X-rays and CT scans have found a small but significant increased lifetime risk [11, 12]. While the benefits of diagnostic X-ray and CT scans outweigh potential individual lifetime risk, their use should be justified and alternatives considered.

We know remarkably little of molecular mechanisms that may be protective or risky for breast cancer after exposure to low-dose ionizing radiation (LDIR). Identification of transcriptomic changes induced by LDIR in mammary tissue will be valuable to elucidate the molecular mechanisms associated with radiation-induced breast cancer. Long noncoding RNAs (lncRNAs), which initially were thought of as transcriptional noise, are emerging as key regulators of a multitude of cellular processes by taking part in epigenetic, transcriptional, and posttranscriptional regulation of gene expression [13, 14]. The lncRNAs have a weaker evolutionary constraint and lower levels of expression compared to the protein-coding transcripts [15, 16] but exhibit more tissue specific expression than the protein-coding genes. Recently, a number of studies have shown that lncRNA expression can be deregulated in human cancers [17, 18]. As the functions of individual lncRNAs in cancer are beginning to be elucidated, they are being categorized and referred to as either tumor suppressor or oncogenic lncRNAs, in the same way as traditional protein-coding cancer genes. However, the relevance of lncRNAs in the cell and tissue response to ionizing radiation has not yet been characterized.

In this study, we used Agilent SurePrint G3 microarrays to profile lncRNA and mRNA from mammary glands of BALB/c mice 2, 4, and 8 weeks after irradiation and of SPRET/EiJ mice 4 weeks after irradiation with 10 cGy of X-radiation. We identified lncRNA and mRNA expression signatures for each time point after irradiation in comparison to sham. Of the total 1338 lncRNAs identified to be differentially expressed after LDIR in either BALB/c or SPRET/EiJ, 1337 had a significantly correlated expression pattern with at least one mRNA that was also differentially expressed after LDIR. Our results indicate lncRNAs may exert a partial or key role in the regulation of coding RNA expression induced by radiation.

## 2. Materials and Methods

### 2.1. Mice and Irradiation

BALB/c and SPRET/EiJ mice were purchased from Jackson Laboratory, housed four per cage under a 12 hr light and 12 hr dark cycle, and fed with Lab Diet 5008 chow and water ad libitum. The mice were irradiated whole body at 8-9 weeks of age to a single dose of 10 cGy using a Precision X-ray Inc RAD320 320 kVp X-ray machine, operated at 300 kV, 2 mA. Mammary tissues were collected for gene expression profile at 2, 4, and 8 weeks after irradiation. All animal experiments were performed at Lawrence Berkeley National Laboratory and the study was carried out in strict accordance with the Guide for the Care and Use of Laboratory Animals of the National Institutes of Health. The animal use protocol was approved by the Animal Welfare and Research Committee of the Lawrence Berkeley National Laboratory.

### 2.2. Expression Profile by Microarray

Total RNA quality and quantity were determined using Agilent 2100 Bioanalyzer and NanoDrop ND-1000. Agilent SurePrint G3 Mouse GE 8x60K Microarrays were used according to the manufacturer's protocol (arrays contained 39,430 Entrez gene RNAs and 16,251 lncRNAs). All processes were done by Ambry Genetics (Aliso Viejo, CA). Microarray data have been deposited at NCBI GEO (accession number: GSE62662).

### 2.3. Data Analysis

Data normalization was performed with GeneSpring GX12.5 (Agilent Technologies). Signal intensities for each probe were normalized to the 75th percentile without baseline transformation. Genes that were differentially expressed between sham and irradiated were identified by the unpaired Student's *t*-test with a *p* value cut-off of 0.05 (*p* value < 0.001 for baseline strain comparison) and a fold-change criteria of more than 1.5.

### 2.4. Correlation Analysis

For each of the 8 experimental treatment groups, the average expression values of the 3 biological replicates were first calculated for each mRNA and each lncRNA. Significantly correlated pairs of mRNA and lncRNA were calculated using a standard permutation test [19]. In brief, for each potential mRNA and lncRNA pair, the 8 mRNA values were randomly rearranged and a correlation coefficient was calculated between the 8 mRNAs and 8 lncRNAs values. Permutations were repeated 10,000 times to derive a distribution of 10,000 correlation coefficients. The *p* value reported in this work represents the percentage of permutations leading to a higher correlation than the original correlation between the 8 mRNAs and 8 lncRNAs values. In other words, the lower the *p* value is, the more likely the mRNA and lncRNA pair is not randomly associated.

### 2.5. Functional Analysis

**Figure 1:**
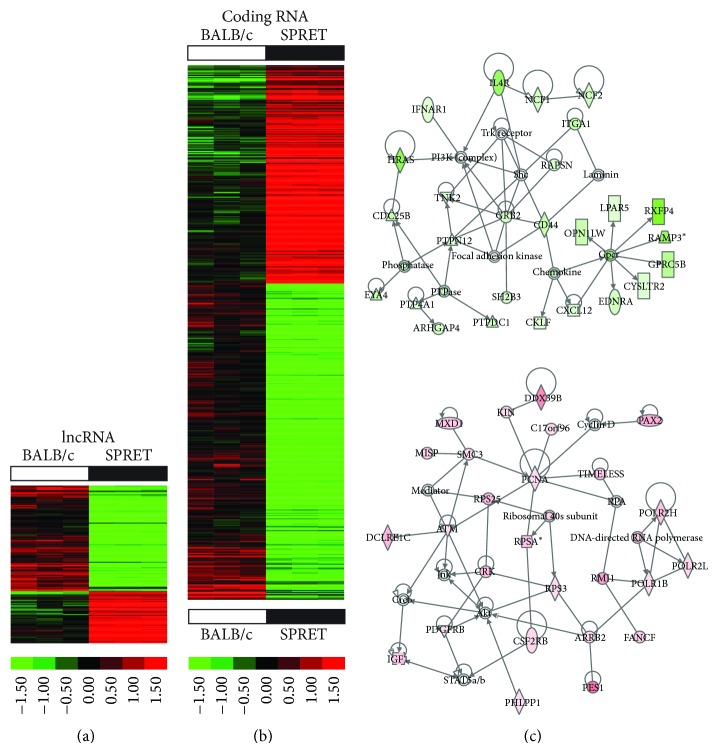
Significant strain differences in lncRNA and coding RNA expression in mammary tissues of BALB/c and SPRET/EiJ mice. (a)-(b) Hierarchical clustering of baseline differences in lncRNA (a) and coding RNA (b) expression in mammary gland tissues of 8-9-week-old BALB/c and SPRET/EiJ mice (fold-change 1.5; *p* value < 0.001). Increased expression indicated in red and decreased expression in green. (c) Gene interaction networks of genes expressed at lower levels in BALB/c mammary tissues when compared to SPRET/EiJ (top) and genes expressed at higher levels in BALB/c compared to SPRET/EiJ (bottom) (see Figure S3 for annotation of molecular shapes used in the networks).

**Figure 2:**
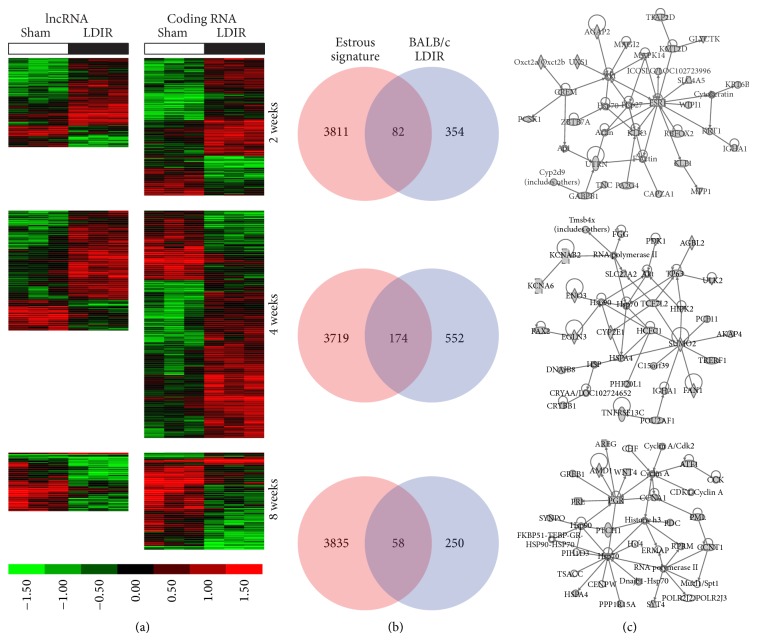
Significant lncRNA and coding RNA expression changes after LDIR exposure in mammary tissues of BALB/c mice. (a) Hierarchical clustering of lncRNAs (left) and coding RNAs (right) differentially expressed (fold-change 1.5; *p* < 0.05) in mammary gland tissues of BALB/c mice at 2, 4, and 8 weeks after sham or LDIR exposure. Increased expression indicated in red and decreased expression in green. (b) Comparison of genes differentially expressed after low-dose radiation at each timepoint with a gene signature containing genes whose expression significantly changes in the mammary gland across the female estrous cycle. (c) The most significant gene interaction networks of estrous cycle independent genes differentially expressed after LDIR at 2, 4, and 8 weeks after exposure (see Figure S3 for annotation of molecular shapes used in the networks).

**Figure 3:**
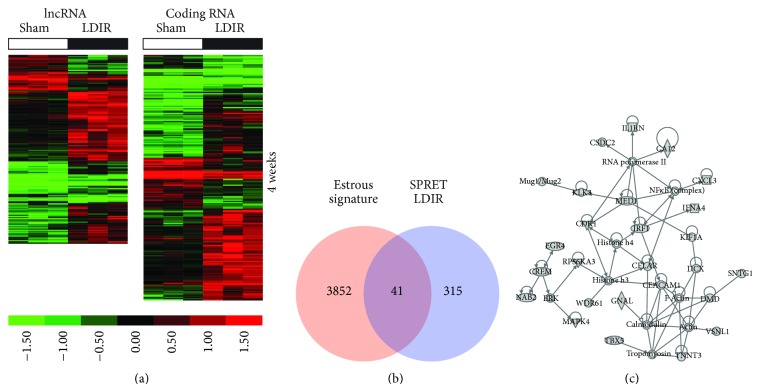
Significant lncRNA and coding RNA expression changes after LDIR exposure in mammary tissues of SPRET/EiJ mice. (a) Hierarchical clustering of lncRNAs (left) and coding RNAs (right) differentially expressed (fold-change 1.5; *p* < 0.05) in mammary gland tissues of SPRET/EiJ mice at 4 weeks after sham or LDIR exposure. Increased expression indicated in red and decreased expression in green. (b) Comparison of genes differentially expressed after low-dose radiation with a gene signature containing genes whose expression significantly changes in the mammary gland across the female estrous cycle. (c) The most significant gene interaction network of estrous cycle independent genes differentially expressed after LDIR (see Figure S3 for annotation of molecular shapes used in the networks).

Gene lists were annotated with biological functions using Ingenuity Pathway Analysis (IPA), KEGG pathway analysis (http://bioinfo.vanderbilt.edu/webgestalt/) and DAVID GO gene ontology (http://david.abcc.ncifcrf.gov/; *p* ≤ 0.05). Annotations for the various shapes used in the IPA networks in Figures 1–3 are shown in Figure S3 in Supplementary Material available online at http://dx.doi.org/10.1155/2015/461038. lncRNA and mRNA correlation networks were generated using Cytoscape.

## 3. Results and Discussion

### 3.1. Differential Expression of lncRNA and mRNA between BALB/c and SPRET/EiJ Mammary Tissues

To identify potential lncRNAs and mRNAs that may determine susceptibility to radiation-induced breast cancer, we profiled two inbred strains of mice with differing genetic susceptibilities: BALB/c mice as more sensitive and SPRET/EiJ as more resistant. BALB/c mice carry two DNA-PKcs polymorphisms with reduced catalytic subunit activity and defective nonhomologous-end-joining of double strand breaks [20]. We identified 195 lncRNAs as upregulated and 95 lncRNAs as downregulated in BALB/c in comparison to SPRET/EiJ (fold-change 1.5; *p* value < 0.001) (Figure 1(a); Table S1). Additionally, 582 mRNAs were upregulated and 402 mRNAs were downregulated in BALB/c in comparison to SPRET/EiJ (fold-change 1.5; *p* value < 0.001) (Figure 1(b); Table S1). Gene ontology analyses of differentially expressed genes between BALB/c and SPRET/EiJ showed significant enrichment for metabolic processes (*p* = 5.9*E* − 06), ion binding (*p* = 3.00*E* − 08), and chemokine signaling (*p* = 0.02) (Figure 1(c); Table S2).

Our analyses identified significant strain differences in gene expression between BALB/c and SPRET/EiJ mammary tissues. We found significant differences in the expression of a number of chemokines including CXCL10, CCL6, and CCL25, which were expressed at higher levels in mammary tissues of the more sensitive and susceptible BALB/c mice and which have previously been associated with breast cancer progression when over expressed [21].

### 3.2. lncRNA and mRNA Expression Signatures of Irradiated Mammary Tissues

To identify lncRNA and mRNA expression changes induced by low-dose ionizing radiation (LDIR), we profiled lncRNA and mRNA expression from mammary glands of BALB/c mice 2, 4, and 8 weeks after irradiation and of SPRET/EiJ mice 4 weeks after irradiation with 10 cGy of X-radiation. For BALB/c mice, a total of 357, 480, and 335 lncRNAs and 550, 911, and 389 coding RNAs were identified to be differentially expressed at weeks 2, 4, and 8 after IR in comparison to sham (fold-change 1.5; *p* value < 0.05), respectively (Figure 2(a); Table S1). For SPRET/EiJ, a total of 327 lncRNAs and 424 mRNAs were identified as differentially expressed at week 4 after irradiation in comparison to sham (fold-change 1.5; *p* value < 0.05) (Figure 3(a); Table S1). Few coding-RNAs and lncRNAs were found to be differentially expressed at different time points (Figure S1(A) and S1(B)) and between BALB/c and SPRET/EiJ (Figure S1(C)).

To determine the biological functions associated with the LDIR response, we excluded genes whose levels fluctuate in the mouse mammary gland across the estrous cycle. We recently mapped transcript-level changes across the estrous cycle in the murine mammary gland using RNA sequencing and defined a comprehensive estrous variable gene signature of 3893 genes whose levels fluctuate in mammary glands of BALB/c mice [22]. Comparison of our mapped LDIR genes in mammary glands of BALB/c and SPRET/EiJ mice with the estrous signature revealed an approximate 20% overlap in BALB/c (Figure 2(b)) and 9% overlap in SPRET/EiJ mice (Figure 3(b)). Nonoverlapping and differentially expressed LDIR genes for each of the time points were then computationally mapped to biological functions, pathways, upstream regulators, and networks. These analyses suggested that the LDIR response signatures in mammary glands of BALB/c mice transitions between time points and is distinct from the LDIR response in SPRET/EiJ mice.

Two weeks after LDIR exposure pathways and biological functions significantly enriched in mammary glands of BALB/c mice compared to sham irradiated mice included chemokine signaling (*p* = 0.01), CCR3 signaling in eosinophils (*p* = 0.05), cellular movement (3.92*E* − 04 < *p* < 3.41*E* − 02), and cell death and survival (1.29*E* − 03 < *p* < 3.41*E* − 02). Gene interaction networks were enriched for tissue and endocrine system injury (Figure 2(c) top panel) and significant predicted upstream regulators (Table S3) include GLI2 (*p* = 5.29*E* − 04) and SATB1 (*p* = 1.98*E* − 03). Similar to the two-week LDIR response, GLI2 was predicted to be an upstream regulator (*p* = 4.52*E* − 03; Table S3). GATA3 and STAT6 were among other significant upstream regulators associated with the four-week low-dose response (Table S3). We furthermore observed that the mammary gland of BALB/c mice four weeks after LDIR was enriched for inflammatory response genes (1.84*E* − 05 < *p* < 1.63*E* − 02), cell movement (3.51*E* − 06 < *p* < 1.45*E* − 02), cell-cell signaling (2.79*E* − 05 < *p* < 1.63*E* − 02), and morphology (1.41*E* − 04 < *p* < 1.60*E* − 02), while gene interaction networks were enriched for lipid metabolism (Figure 2(c) middle panel). Interestingly, similar responses were observed in mammary glands of SPRET mice at 4 weeks after LDIR including inflammatory response functions (1.69*E* − 03 < *p* < 4.49*E* − 02), cell-cell signaling (5.54*E* − 04 < *p* < 4.49*E* − 02), and morphology (8.31*E* − 05 < *p* < 4.49*E* − 02), suggesting that the functional response is similar across strains and is independent of the gene transcript response. At 8 weeks after LDIR we observed downregulation of genes involved in mammary gland development including progesterone receptor, prolactin, amphiregulin, and WNT4 (Figure 2(c) bottom panel; Table S3).

### 3.3. Significantly Correlated lncRNA and mRNA Expression Patterns

**Figure 4:**
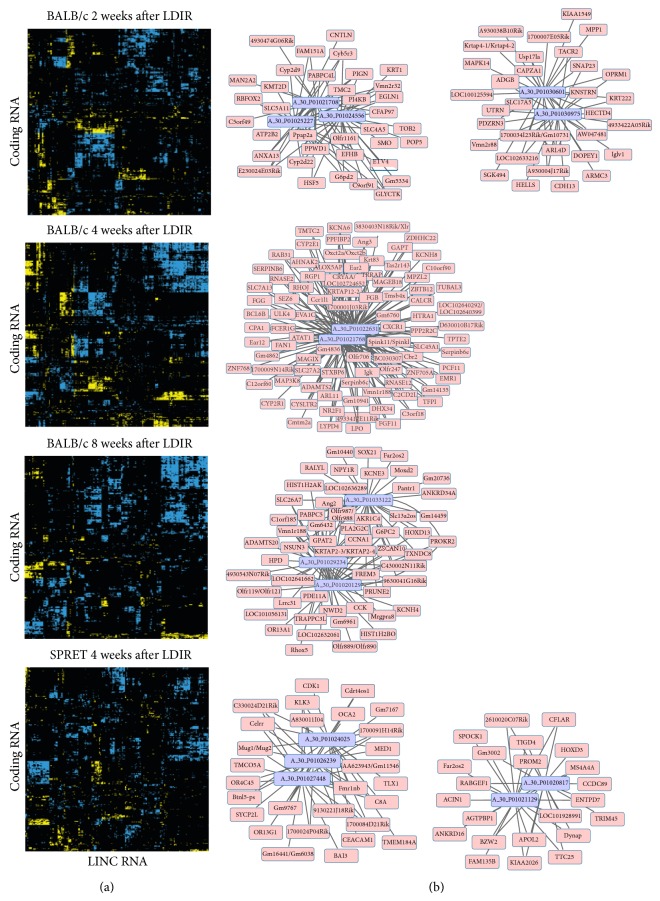
Correlated lncRNA and coding RNA expression in mammary tissues after low-dose radiation exposure. (a) Correlation graphs of the average expression values from each experimental treatment group for each mRNA and lncRNA differentially expressed after LDIR at each of the four timepoints. Positive and negative correlations are indicated in blue and yellow, respectively. (b) Representative examples of networks of lncRNAs (purple) significantly correlated in expression with mRNAs (pink).

To identify lncRNAs potentially regulating the expression of coding RNAs in response to radiation, correlation coefficients on expression data were calculated for each coding RNA and lncRNA that were identified as differentially expressed after LDIR. A permutation-based algorithm was then used to determine which correlations were statistically significant (*p* < 0.05; Table S4). We observed that nearly all LDIR modulated lncRNAs were correlated with at least one of the LDIR modulated coding mRNAs (Figure 4(a)). To determine whether these correlations were driven by estrous variations, we only considered genes whose expression levels were not overlapping with our previously determined estrous signature (Figure 2(b)). Again, we observed that nearly all differentially expressed lncRNAs were correlated with at least one differentially expressed mRNA suggesting that estrous cycling does not affect the strong correlation between lncRNA and mRNA expression after LDIR. To test the robustness of these correlations, we compared the number of lncRNAs associated with at least one mRNA at three different *p* values (*p* < 5*E* − 02, *p* < 5*E* − 03, and *p* < 5*E* − 04). At *p* < 5*E* − 02 or *p* < 5*E* − 03, nearly all (97–100%) differentially expressed lncRNAs were found to be correlated with at least one mRNA (Table S5). At *p* < 5*E* − 04, corresponding to a correlation coefficient >0.9, we still observed a significant fraction (63–81%; Table S5) of lncRNAs correlated with mRNAs. Representative correlation networks of lncRNAs are shown in Figure 4(b) (*p* < 5*E* − 03) for each of the timepoints. We furthermore observed that the same lncRNA correlates with different gene sets across different time points (Figure S2). Taken together these data show that LDIR induces coordinated changes in lncRNA and mRNAs and suggests a critical role for lncRNAs in mediating the low-dose radiation response.

## 4. Conclusions

In this study, we demonstrate that genetic background strongly influences the expression of lncRNAs and their response to low-dose radiation by transcriptomic analysis of mouse mammary glands using microarrays that contain both lncRNAs and coding RNAs. We have identified a number of lncRNAs that are significantly changed after exposure to LDIR at three different timepoints after radiation exposure. These lncRNAs have the potential to be surrogate indicators of tissue radiation responses. Moreover, the changes in the expression of lncRNAs are significantly correlated with the expression of coding RNAs, suggesting that lncRNAs may coordinate the tissue response to radiation via regulation of coding mRNAs. However, the specific regulatory mechanism of this control requires further investigation, and knock-out and overexpression of the lncRNA genes in mice and other model systems should be performed to increase our understanding of the regulatory mechanisms in response to LDIR.

## Supplementary Material

Supplementary Data: The supplementary data include supplementary tables (S1 to S4) and supplementary figures (S1 to S3).
Table S1: LncRNA and mRNA mammary tissue expression signatures of strain differences and after LDIR.
Table S2: Baseline gene expression differences in mammary gland tissue of BALB/c and SPRET/EiJ are associated with diverse biological funcitons.
Table S3: Upstream transcriptional regulators associated with low-dose radiation response in mammary glands of BALB/c and SPRET/EiJ mice.
Table S4: Correlations between lncRNA and coding RNA expression in BALB/c and SPRET/EiJ mammary tissues after LDIR exposure.
Figure S1: LncRNA and coding RNA expression changes in mammary tissues of BALB/c and SPRET/EiJ mice. For each time-point, differentially expressed lncRNA (A) or coding RNAs (B) between sham and low-dose irradiated glands were identified by the unpaired Student's t-test with a p-value cut-off of 0.05 and a fold change criteria of more than 1.5. C. Comparison of differentially expressed lncRNAs (left) and coding RNAs (right) in BALB/c and SPRET/EiJ mice four weeks after LDIR exposure. (BC=BALB/c; SPR=SPRET/EiJ; lnc=lncRNA; cd=coding gene).
Figure S2: LncRNA and coding RNA expression levels are correlated in mammary tissues after low dose radiation exposure. Representative examples of networks of the same lncRNAs (pink) significantly correlated in expression with mRNAs (purple) after LDIR in mammary tissues of BALB/c mice at 2 and 4 weeks after exposure, 4 and 8 weeks after exposure and 2 and 8 weeks after exposure.
Figure S3: Annotation of the molecular shapes used in gene interaction networks. Graphical representation of commonly used molecular shapes in interaction networks generated by Ingenuity Pathway Analysis.
